# Resistance Profile of Osimertinib in Pre-treated Patients With EGFR T790M-Mutated Non-small Cell Lung Cancer

**DOI:** 10.3389/fonc.2021.602924

**Published:** 2021-05-06

**Authors:** Maria Gabriela O. Fernandes, Catarina Sousa, Maria Jacob, Leonor Almeida, Vanessa Santos, David Araújo, Hélder Novais Bastos, Adriana Magalhães, Luís Cirnes, Conceição Souto Moura, Henrique Queiroga, Natália Cruz-Martins, Venceslau Hespanhol

**Affiliations:** ^1^Pulmonology Department, Centro Hospitalar e Universitário de São João, Porto, Portugal; ^2^Faculty of Medicine, University of Porto, Porto, Portugal; ^3^Institute of Molecular Pathology and Immunology of the University of Porto (IPATIMUP), Porto, Portugal; ^4^Institute for Research and Innovation in Health (i3S), University of Porto, Porto, Portugal; ^5^Escola Superior de Saúde (ESS), Instituo Politécnico do Porto (IPP), Porto, Portugal; ^6^Pathology Department, Centro Hospitalar Universitário de São João, Porto, Portugal; ^7^Laboratory of Neuropsychophysiology, Faculty of Psychology and Education Sciences, University of Porto, Porto, Portugal

**Keywords:** non-small cell lung cancer, EGFR T790M mutation, osimertinib, resistance, real-world data, next generation sequencing

## Abstract

**Background:** Osimertinib efficacy in pre-treated patients with epidermal growth factor receptor (EGFR) T790M-mutated non-small cell lung cancer (NSCLC) has been demonstrated in clinical trials, but real-world data, particularly regarding resistance profile, remains limited. This study aims to analyze the resistance mechanisms acquired after treatment with Osimertinib.

**Methods:** Clinical outcomes and molecular results from re-biopsies at the time of osimertinib progression of EGFR T790M-mutated NSCLC patient were analyzed.

**Results:** Twenty-one patients with stage IV adenocarcinoma were included [median 69 years; 57.1% female; 85.7% never-smokers; 23.8% ECOG performance status (PS) ≥2]. Median PFS and OS were 13.4 (95% CI: 8.0–18.9) and 26.4 (95% IC: 8.9–43.8) months, respectively. At the time of analysis, 10 patients had tumor progression (47.6%). T790M loss occurred in 50%, being associated with earlier progression (median PFS 8.1 vs. 21.4 months, *p* = 0.011). Diverse molecular alterations were identified, including C797S mutation (*n* = 1), PIK3CA mutation (*n* = 2), MET amplification (*n* = 1), CTNNB1 mutation (*n* = 1), and DCTN1-ALK fusion (*n* = 1). Histological transformation into small cell carcinoma occurred in one patient.

**Conclusions:** This real-world life study highlights the relevance of re-biopsy at the time of disease progression, contributing to understand resistance mechanisms and to guide treatment strategies.

## Introduction

Patients with advanced non-small-cell lung cancer (NSCLC) with activating mutations in the epidermal growth factor receptor (EGFR) gene are eligible for EGFR tyrosine kinase inhibitors (TKIs). Despite the high response rates to first-line TKIs and a median progression-free survival (PFS) of 10–14 months (1–8), the disease ultimately progresses. In about 50–60% of patients, the acquired mechanism of resistance to first-line TKIs is a p.Thr790Met point mutation (T790M) in the EGFR gene. This mutation increases the receptor affinity for ATP binding, drastically reducing the drug activity (9–12).

Osimertinib is an irreversible EGFR-TKI that is selective for both EGFR and T790M resistance mutations (13). In the Phase III AURA3 trial (AZD9291 vs. Platinum-Based Doublet-Chemotherapy in Locally Advanced or Metastatic Non-Small Cell Lung Cancer), osimertinib was superior to platinum therapy plus pemetrexed in patients with T790M whom the disease progressed during first-line EGFR-TKI therapy with a median PFS of 10.1 months, and objective response rate (ORR) of 71% (14). Moreover, osimertinib had significant efficacy in patients with central nervous system (CNS) metastases (15). Recently, in the Phase III FLAURA trial (AZD9291 vs. Gefitinib or Erlotinib in Patients With Locally Advanced or Metastatic Non-small Cell Lung Cancer), Osimertinib was also superior in first-line (16).

Despite the survival data and response rates for osimertinib, acquired resistance, unfortunately, occurs after about 10 months (17). The mechanisms that determine disease progression are heterogeneous and not fully understood, including on-target EGFR-dependent and off-target independent mechanisms. EGFR-dependent mechanisms include new tertiary mutations, like the exon 20 C797S mutation, EGFR amplification or T790M disappearance. EGFR independent mechanisms can occur with bypass pathway activation, such as erb-b2 receptor tyrosine kinase 2 (HER2) and MET amplification, PIK3CA activating mutations, PTEN deletion, RAS mutations, fusions affecting anaplastic lymphoma kinase (ALK), and RET and others. There is also the possibility of phenotypic alteration, such as the transformation in small-cell lung cancer (SCLC) (18–20).

Treatment approaches for patients progressing from third-generation EGFR TKIs have not been clearly established. However, in case of disease progression without targeted therapy available, chemotherapy is still indicated and maintaining osimertinib beyond progression, with or without adjunctive radiotherapy, can be a useful option (21, 22). Although, a considerable amount of data is published on 3rd generation EGFR-TKIs, real-world data is limited. In this sense, this study aims to analyze the resistance profile of Osimertinib in a T790M EGFR-mutated population.

## Materials and Methods

### Study Design

A retrospective analysis of T790M-mutated NSCLC patients treated with osimertinib, at the Centro Hospitalar e Universitário de São João (CHUSJ), Porto, Portugal, was performed. This study was conducted under the Declaration of Helsinki and was approved by the Ethics Committee of CHUSJ (243/20).

Eligible patients were required to have histologically confirmed stage IV NSCLC (based on TNM staging AJCC 8th edition), with an activating EGFR mutation, treated with osimertinib after progression with at least one 1st or 2nd generation TKI and with confirmed EGFR T790M mutation identified by re-biopsy at the time of progression. Patients initiated osimertinib between August 2016 and April 2019. Last data analysis was performed on 30 April 2020.

Patient demographics and clinical features, tumor histology, disease stage, lines of treatment received before osimertinib, and pattern of progression were recorded.

Molecular analyses from initial biopsies and re-biopsies at osimertinib progression were reviewed. Digital protein chain reaction (PCR) was used for EGFR T790M detection, and next-generation sequencing (NGS) at the time of progression was performed using a validated amplicon-based NGS (Oncomine^TM^ Focus Assay, ThermoFisher). These assays allow the analysis of targeted regions in EGFR, KRAS, NRAS, BRAF, MET, HER2, HER4, PIK3CA, and ALK genes plus the detection of ALK, ROS1, RET and NTRK (1, 2, and 3) gene fusions.

### Statistical Analysis

Most analysis was descriptive. Categorical variables are presented as relative frequencies and percentages, and continuous variables as median, interquartile range (IQR) and minimum and maximum values. Kaplan-Meier actuarial curves analysis was used to estimate OS, PFS and time to treatment discontinuation (TTD) for the entire cohort. Group comparisons were performed using the Mann-Whitney test. The significance level assumed was 0.05. All statistical analyses were performed using the Statistical Package for Social Sciences (SPSS, IBM Corp, Chicago, IL, USA) software, version 25.0.

## Results

### Patients' Characteristics

**Figure 1:**
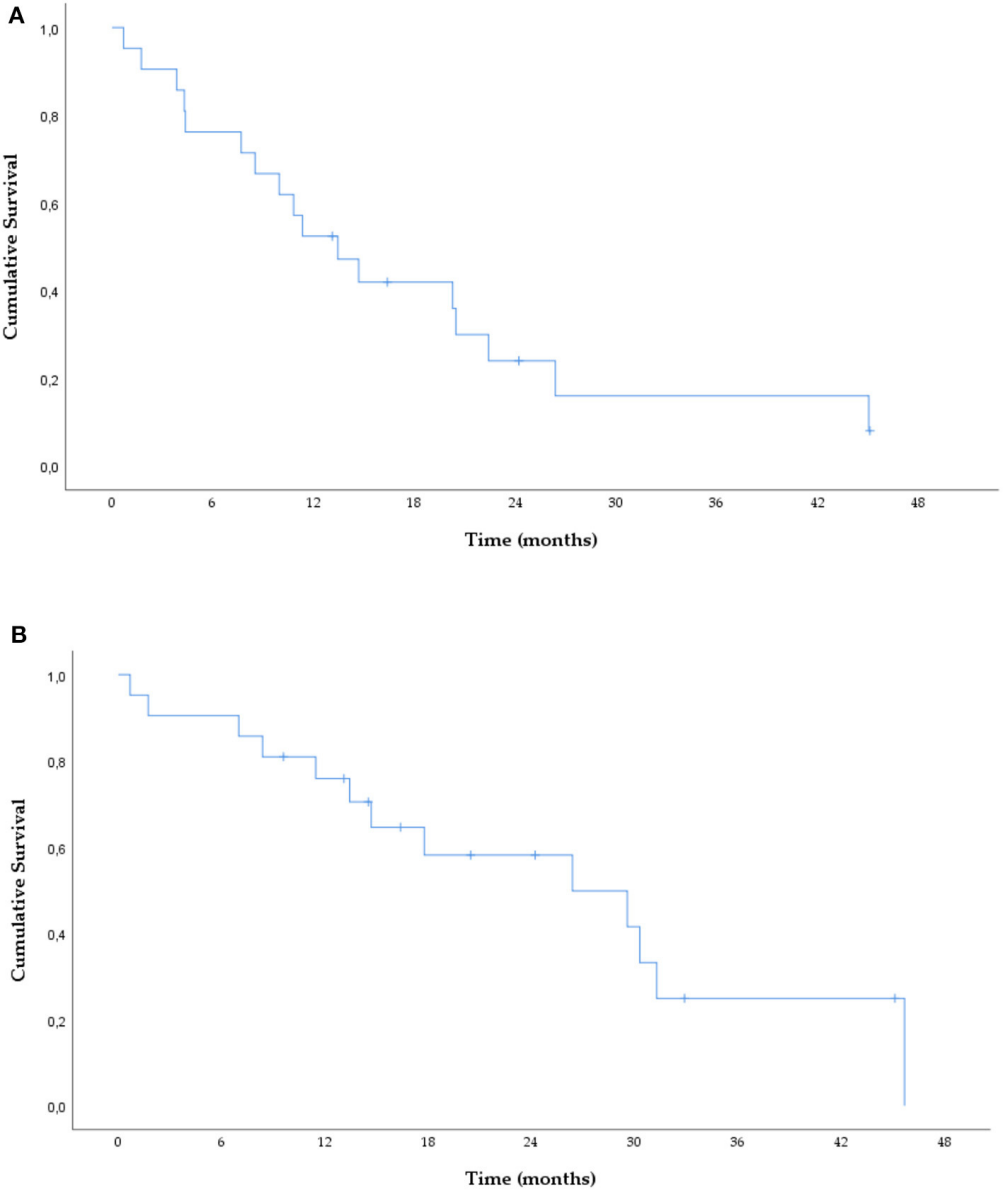
PFS **(A)** and OS **(B)** under osimertinib treatment.

Twenty-one patients treated with osimertinib were included (Table 1), with median age of 69 (range 39–84) years, 12 (57.1%) were female, mostly never-smokers (*n* = 18; 85.7%). Of note, 13 (61.9%) patients were ≥65 years, and 5 (23.8%) had an ECOG performance status (PS) ≥2. All patients were diagnosed with stage IV adenocarcinoma [IVA *n* = 9 (42.9%); IVB *n* = 12 (57.1%)]. Exon 19 deletion and exon 21 L858R mutations were present, at initial biopsy, in 17 (85%) and 3 (15%) cases, respectively. The T790M mutation was detected by tissue biopsy in eight (38.1%), liquid biopsy in five (23.8%), and by both in 8 (38.1%) patients. Osimertinib was given as 2nd line treatment in 13 cases (61.9%), after a 1st or 2nd generation EGFR-TKI, and in 3rd or more line in eight cases (38.1%). The best ORR was 52.7% (nine partial responses; one complete response) and DCR 89.5% (seven with stable disease), respectively. Median PFS was 13.4 (95% CI: 8.0–18.9) months (Figure 1A). Of the 17 cases with an objective response/disease control, 10 subsequently progressed and underwent re-biopsy. There were eight patients with oligo-progression (80%) and two with systemic progression (20%). The main sites of progression were bone (*n* = 4), lung (*n* = 4), pleura (*n* = 2), CNS (*n* = 1), and liver (*n* = 1). Thirteen (61.9%) patients died, with a median OS since osimertinib initiation of 26.4 months (95% IC: 8.9–43.8) (Figure 1B).

**Table 1:** Baseline patients' characteristics.

**Characteristics**	***n* (%)**
Age, years	
Median (range)	69 (39–84)
Gender	
Female	12 (57.1)
Male	9 (42.9)
Performance status	
0–1	16 (76.2)
≥2	5 (23.8)
Smoking status	
Never	18 (85.7)
Smoker/former smoker	3 (14.3)
Type of EGFR sensitizing mutation	
Exon 19 deletion	18 (85.7)
L858R (exon 21)	3 (14.3)
Stage	
IVA	9 (42.9)
IVB	12 (57.1)
Metastasis	
CNS	3 (14.3)
Extra-thoracic	13 (61.9)
Previous treatment	
1	13 (61.9)
≥2	8 (38.1)
Previous TKIs	
1st generation (erlotinib/gefitinib)	18 (85.7)
2nd generation (afatinib)	1 (4.8)
Sequential TKIs	2 (9.5)

*CNS, central nervous system*.

Osimertinib was well-tolerated, with only two cases reporting grade ≥3 AE, corresponding to pneumonitis resolved with definite discontinuation (9.5%) and corticosteroids treatment.

### Post-osimertinib Resistance Profile and Progression Treatment

**Figure 2:**
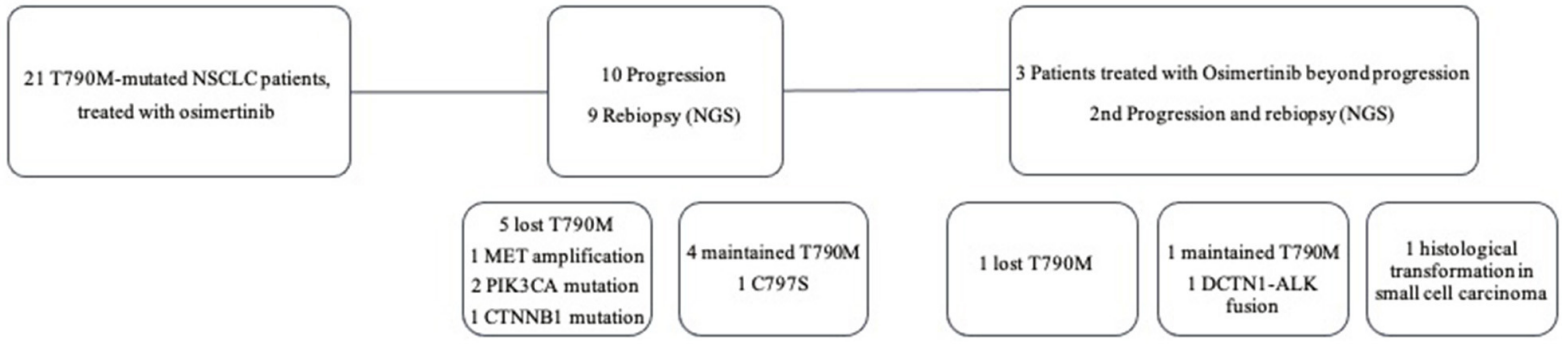
Treatment and resistance profile of NSCLC patients post-osimertinib progression.

At the time of analysis, 10 patients had tumor progression (47.6%), and the resistance profile is summarized in Figure 2 and Table 2.

**Table 2:** Resistance profile of osimertinib.

**Group**	**Initial biopsy**	**1st re-biopsy**	**PFS (months)**	**Post-progression treatment**	**Treatment response**	**2nd re-biopsy**	**2nd post-progression treatment**	**Treatment response**
					**Post-progression PFS (months)**			**Post-progression PFS 2 (months)**
Loss of T790M	T790M Exon 19 del	Exon 19 del MET amp	3.8	ChT	Death 1.5			
	T790M Exon 19 del	Exon 19 del PIK2CA mut CTNNB1 mut	7.7	2nd generation TKI (afatinib)	Progression 2.0			
	T790M Exon 19 del	Exon 19 del	8.5	2nd generation TKI (afatinib)	Death 0.5			
	T790M Exon 19 del	Exon 19 del	4.3	ChT	Stable disease 3.8			
	T790M L858R	L858R PIK3CA mut	10.8	ChT	Stable disease 3.5			
Maintained T790M	T790M Exon 19 del	T790M Exon 19 del	20.3	Osimertinib LAT	Progression 3.2	Small cell carcinoma	ChT	Death 3.1
	T790M Exon 19 del	T790M Exon 19 del	11.3	Osimertinib LAT	Progression 13.1	Exon 19 del	ChT	Death 5.6
	T790M Exon 19 del	T790M Exon 19 del C797S	22.4	ChT	Progression 4.8			
	T790M^*^ Exon 19 del	-	20.5	Osimertinib	Progression 5.0	T790M Exon 19 del DCTN1/ALK fusion	ALK inhibitor (crizotinib)	Partial response 5.0
	T790M Exon 19 del	T790M Exon 19 del	45.90	BSC	Death 0.6			

* *Patient with 2 re-biopsies (1st: maintained T790M; 2nd: lost T790M)*.

A total of 12 re-biopsies were analyzed among the 10 patients who progressed (Figure 2). Molecular testing was performed in all cases, 10 on tissue biopsy (83.3%), including 8 computed tomography (CT)-guided percutaneous core needle biopsy (PCNB), one ultrasound-guided liver biopsy and one CT-guided soft tissue biopsy, and two (16.7%) on liquid biopsy. The patients that performed liquid biopsy presented no clinical conditions for tissue biopsy (*n* = 1) or inaccessible disease (*n* = 1).

T790M mutation loss occurred in 50% of cases (*n* = 6), but other molecular changes were also found among this group, including PIK3CA mutation (*n* = 2), MET amplification (*n* = 1), and CTNNB1 mutation (*n* = 1). In the T790M-persistent group, a patient presented a newly exon 20 C797S mutation and another a DCTN1-ALK fusion (*n* = 1). Histological transformation in SCLC occurred in one patient.

T790M mutation loss was associated with earlier progression [PFS: median 8.1 (range: 3.8–11.3) vs. 21.4 months (range: 20.3–45.0), *p* = 0.011] and worse OS [median 13.0 (range: 7.0–30.3) vs. 32.1 months (range: 29.6–45.7), *p* = 0.019].

Of the 10 progressing patients, nine received at least one subsequent treatment, three received osimertinib beyond progression (33.3%), two of them in association with local ablative treatment (LAT), and six initiated a new treatment line [ChT (*n* = 4); another EGFR-TKI (*n* = 2)]. One patient received best supportive care (BSC). The patient with DCTN1-ALK fusion started crizotinib, presenting a partial response at the 3-month CT evaluation. The three patients who received osimertinib beyond progression had a new re-biopsy at the time of 2nd progression (Figure 2), and all received a new treatment line (2 ChT). The median post-progression PFS (ppPFS) was 5.0 months (range: 3.2–13.1; all cases with progression). The ppPFS of those who received a new treatment line was 2.7 months (range: 0.5–4.8; 2 cases without progression), *p* = 0.12.

## Discussion

Randomized controlled trials are the gold standard in clinical research. Still, real-world data is essential to verify the effectiveness, safety, application of treatment in the general population and to understand the patient's evolution in daily clinical practice. This is the first report of osimertinib in pre-treated EGFR T790M-mutated NSCLC patients in our population, focusing on the resistance mechanisms, and progression profile.

Identifying the resistance profile is critical in selecting the appropriate treatment after osimertinib, as several biological mechanisms of acquired resistance have been identified. To fully capture the diversity of resistance mechanisms is essential to repeat a biopsy to obtain the best possible sample that harbors the alteration responsible for progression with the less invasive and safer technique. However, obtaining tissue samples from patients experiencing progressive disease after EGFR-TKI failure remains a challenge. Rate of patients submitted to re-biopsy ranges from 50 to 60% in different series (23–25). With the analysis of circulating tumor DNA (ctDNA), liquid biopsy is a promising technique considering its invasiveness, repeatability, and accessibility. Some studies proved the role of ctDNA based assays to detect EGFR activations mutations and the T790M. In the osimertinib progression setting, in AURA3 trial, ctDNA genomic profile detected several resistance mechanisms, including MET amplification (26). Nevertheless, the use of cfDNA presents some limitations and challenges, especially considering the occurrence of false-negative results associated with the absence or low DNA of tumoral origin present on plasma or analytical limitations and to the difficulty to detect small cell-transformation. In the setting of EGFR progressive disease, both tissue and liquid assays, are complementary.

Comprehensive NGS panels help to define the genomic diversity of resistance mechanisms and are particularly important in this setting, where there is no single alteration to detect.

In this group, concerning osimertinib efficacy, ORR was 52.7%, median PFS 13.4 and median OS 26.4 months, similar to data from other real-world studies. All patients underwent a new biopsy at the time of progression, mainly tissue re-biopsy, and two liquid biopsies. All samples were evaluated with a targeted gene panel NGS.

We found that T790M mutation loss is common in Osimertinib-resistant cases (50%), consistent with previous studies (21, 27–30). Also, T790M mutation loss was associated with a shorter median PFS, which agrees with a previous study in which acquired resistance to osimertinib mediated by T790M mutation loss was associated with early progression, lower PFS and shorter TTD (28).

Molecular analyses from the AURA 3 trial revealed the presence of acquired EGFR mutations in 21% of patients, most commonly a new exon 20 point mutation C797S (14%) (26). In our series, only one patient acquired the C797S mutation. Most of the molecular alterations found were in EGFR-independent pathways, two PIK3CA mutation, one MET amplification, one CTNNB1 mutation, and one DCTN1-ALK fusion. In one patient occurred histological transformation into SCLC, a mechanism previously described in other studies (18, 20, 31).

Until today, no specific drug has been approved for the treatment of Osimertinib resistant patients, and a plethora of strategies are being explored. Rechallenge with 1st/2nd generation TKIS for C797S occurring in *trans* can be an option (32). Innumerous therapeutic combinations between osimertinib and antiangiogenics can be an option to overcome EGFR-dependent resistance mechanisms (33). Combination of Osimertinib and other inhibitors can help overcome resistance mediated through alternative kinase activation, as MET, MEK, and BRAF inhibitors (34–36). For most patients, platinum-based doublet chemotherapy is the only available option.

Regarding resistance to osimertinib, oligo-progression is frequent, being present in our series in more than 2/3 of patients. Schmid et al. (37) also reported this finding in 73% of cases. In this situation, LATs and osimertinib continuation beyond progression can be beneficial (37). In about one-third of cases, osimertinib treatment was continued beyond progression, with a longer ppPFS than patients who started a new treatment line, although not significant (5.0 vs. 2.0 months, *p* = 0.22). In the remaining cases, a new treatment line was started, mostly ChT. Two patients who lost the T790M and maintained the exon 19 deletion were treated with 2nd generation TKI afatinib, with a poor outcome. Crizotinib was initiated in a patient with DCTN1-ALK fusion with partial response.

Although, being a single center, retrospective study with small sample size, it illustrates the feasibility and relevance of performing re-biopsies, and NGS to study the resistance mechanisms at the time of progression, opening the window for new therapeutic strategies as demonstrated.

## Conclusion

Re-biopsy at the time of disease progression is feasible outside clinical trials, being of extreme usefulness to understand the underlying resistance mechanisms, to guide treatment strategies and, consequently, contributing to increase patient's survival.

## Data Availability Statement

The raw data supporting the conclusions of this article will be made available by the authors, without undue reservation.

## Ethics Statement

The studies involving human participants were reviewed and approved by CHUSJ (approval n° 243/20).

## Author Contributions

CS and MGOF: conceptualization and writing—original draft preparation. CS, MGOF, and NC-M: methodology. CS and NC-M: formal analysis. CS, MJ, and LA: investigation and data curation. CS, MGOF, MJ, LA, and NC-M: writing—review and editing. VH and HQ: supervision. All authors have read and agreed to the published version of the manuscript.

## Conflict of Interest

The authors declare that the research was conducted in the absence of any commercial or financial relationships that could be construed as a potential conflict of interest.
